# Regulatory roles of an atypical ubiquitin ligase UBE2O in orphans of multiprotein complexes for degradation

**DOI:** 10.3906/biy-2106-63

**Published:** 2022-02-24

**Authors:** Yi LV, Feiyue XING

**Affiliations:** Department of Immunobiology, Institute of Tissue Transplantation and Immunology, Jinan University, Guangzhou, China

**Keywords:** UBE2O, SMAD6, TRAF6, BAP1, AMPKa2, MLL

## Abstract

UBE2O as an atypical ubiquitin-conjugating enzyme possesses an E2–E3 hybrid enzyme activity. It can regulate substrate levels or transcriptional activities by cooperating with other E3 ubiquitin ligases or forming homomeric complexes displaying intrinsic E2 and E3 activities. UBE2O controls the quality of cell proteome including protein degradation, modification, transport and location. Recent studies reveal that UBE2O plays a vital role in intracellular protein ubiquitination processes by regulating BMP/SMAD, TRAF/NF-κB, mTOR/HIF1a and IL-1β/IRAK4 signaling pathways, c-Maf stability and BAP1 subcellular location, which is proposed as a quality control supervisor of multiprotein complexes for degradation. Its abnormality leads to a variety of physical activity disorders and even occurrence of cancer. UBE2O is entirely distinct in molecular structure and functions from other E2 ubiquitin ligase. Exploring and elucidating regulatory mechanism of UBE2O may identify novel crucial molecular targets so as to pave therapeutic approaches for ubiquitination-associated metabolic disorders and diseases. Here, we particularly feature regulatory pathways of UBE2O in orphans of multiprotein complexes for degradation and its potential application.

## 1. Introduction

Ubiquitination is a process of ubiquitin covalent attachment to target proteins, including three main steps: activation, conjugation and ligation, which regulates protein activity, degradation, cellular location and protein-protein interaction (Cai et al., 2017; Tsuchida et al., 2017). There are several types of substrate ubiquitination, such as monoubiquitination, multimonoubiquitination and polyubiquitination. The anomalous ubiquitin pathway has been involved in the pathogenesis of many diseases and genetic disorders. Ubiquitination requires the cooperation of E1s (ubiquitin activating enzymes), E2s (ubiquitin conjugating enzymes) and E3s (ubiquitin ligases). Nowadays, over 35 E2 enzymes have been identified in the human, but their functions still need to be clarified (Van and Timmers, 2010).

**Figure 1:**
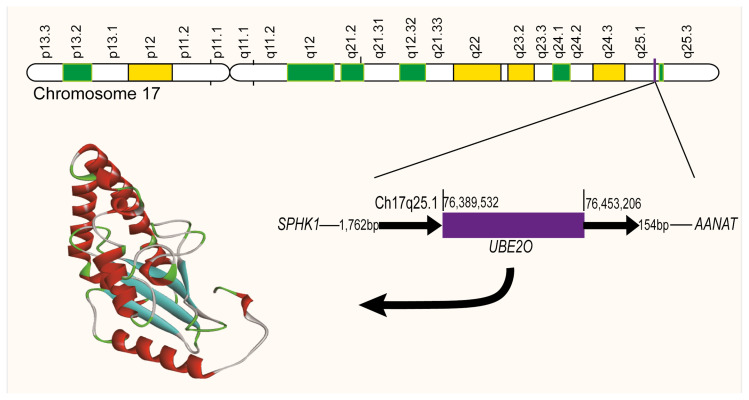
Structure and its position of UBE2O on the chromosome. The genomic location of UBE2O is on human 17q25.1 and its whole length is 63.7 kb. The human UBE2O cDNA length is 4878 nucleotides encoding 1138 amino acids which contain a 56 amino acid conservative residue UBC domain. UBE2O is E2/E3 hybrid ubiquitin-protein ligase that possesses both E2 and E3 ligase activities and mediates monoubiquitination of target proteins.

Ubiquitin-conjugating enzyme E2O (UBE2O) is also named as E2-230K which was first purified from rabbit reticulocytes in 1989 (Klemperer et al., 1989). Yokota T and colleagues identified UBE2O located in 17q25.1 via YAC and PAC genomic clones (Yokota et al., 2001). UBE2O is an atypical E2 ubiquitin enzyme containing an UBC domain with 56 conserved amino acid residues and it also possesses E3 activity (Figure 1). Studies have shown that UBE2O N-terminal portion can selfinteract with C-terminal portion and directly incurs ubiquitination of several substrate in the presence of only the E1 enzyme such as BAP1, SMAD6, AMPKa2, c-Maf, MLL and BMAL1 (Zhang et al., 2013; Mashtalir et al., 2014; Liang et al., 2017; Vila et al., 2017; Xu et al., 2017). It also displays functionally regulating activity that is independent of its E2 activity (Zhang et al., 2013). Unlike most members of E2s, molecular weight of UBE2O (140 kDa) is much larger than other E2 ubiquitin-conjugation enzymes (14-35KDa). It is expressed in a great number of organs and tissues, but predominantly in skeletal muscle, brain and heart tissues (Yokota et al., 2001; Zhang et al., 2013). UBE2O also show high-expression during the reticulocyte period of erythroid differentiation, which is believed to play a critical role in the terminal erythroid differentiation. Markson et al. found through yeast two-hybrid screens that more than 100 E3 ubiquitin ligases have the ability to interact with UBE2O.

It was further found that UBE2O can modulate expressions of ribosomal proteins RPL29, RPL35 and RPL23A, and nonribosomal proteins DDX5, NOL12, NOP16 and PTRF (Nguyen et al., 2017). It affects cell proliferation via regulating BAP1, FOXK1, FOXK2 and KDM1B levels (Mashtalir et al., 2014), and cell cycle via regulating ALDOA, EGR1, GNA15, LGALS1, PE2RY11 and SREBF1 expressions (Liang et al., 2017). Zhang et al. reported that UBE2O also impacts activities of transcriptional factors TRAF6, NF-κB, etc. (Zhang et al., 2013; Ullah et al., 2019). In addition, UBE2O can function in BMP7-induced adipogenesis via monoubiquitination of SMAD6 and also in chromatin remodeling by ubiquitinating the tumor-suppressive DUB BAP1 (Hormaechea-Agulla et al., 2018). However, the functions of UBE2O remain mostly unknown (Markson et al., 2009). Recently, several in vitro and in vivo studies demonstrated that UBE2O is implicated in several cellular pathways (Table). In this paper, we feature current literatures about possible roles of UBE2O in cellular signal transductions to provide a novel insight into pathogenesis of some clinical disorders and pathological processes.

## 2. UBE2O augments BMP signaling by monoubiquitination of SMAD6

**Figure 2:**
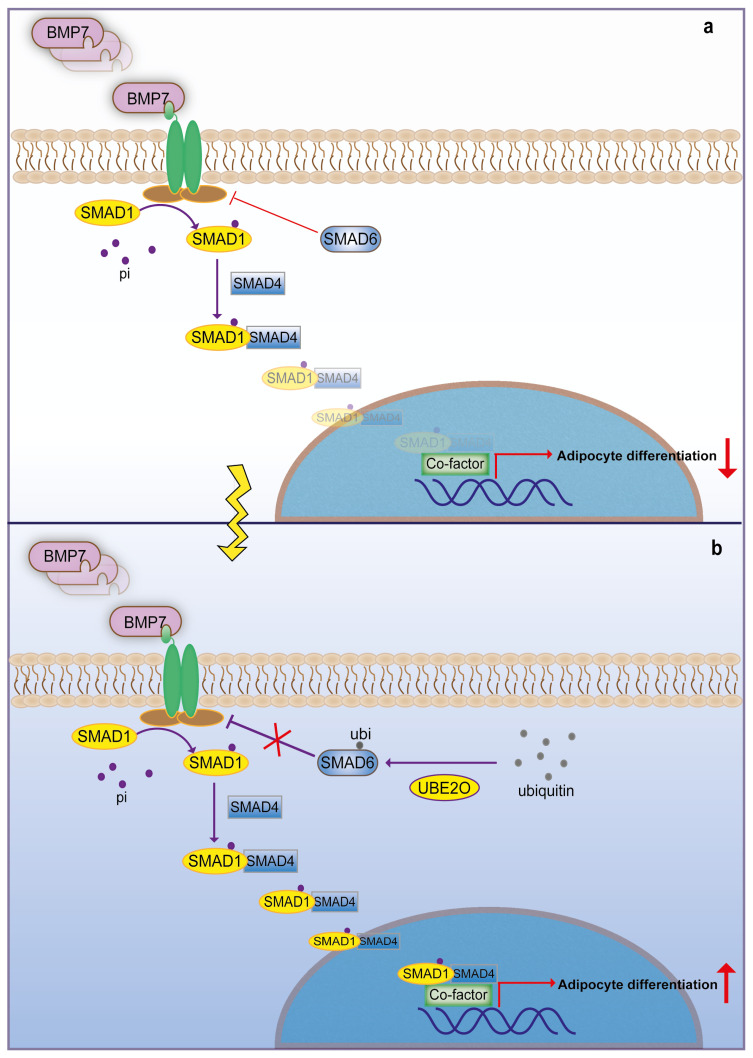
UBE2O augments BMP signaling by monoubiquitination of SMAD6. a. SMAD6 negative feedback regulates BMP-induced adipocyte differentiation. b. UBE2O induces the monoubiquitination of SMAD6 to strengthen the adipocyte differentiation which is activated by BMP signaling cascade.

Bone morphogenetic proteins (BMPs) have been implicated in cell proliferation, differentiation and apoptosis, which were identified on the basis of their ability to promote the formation of bone and cartilage (Miyazono et al., 2010; Plouhinec et al., 2011). BMPs induce a type II receptor binding to a type I receptor to form a heteromeric complex, activating a type I receptor (also named activin receptor-like kinases, ALKs). Activated ALKs deliver the intracellular signal to activate receptor-regulated SMADs (R-SMADs), such as SMAD1/5/8. Then, phosphorylated R-SMADs interact with common-mediator SMAD (co-SMAD, i.e. SMAD4) to form heteromeric complexes and translocate into the nucleus to regulate target genes. SMAD6 is one of the BMP signaling target genes, which plays a role in feedback regulation (Itoh and Ten, 2007; Sieber et al., 2009). Whole proteomic tandem affinity purification assay preformed in HEK293T cells showed that UBE2O interacts with SMAD6, which has been proved by cotransfection of labeled proteins. SMAD6 monoubiquitination by UBE2O attenuated binding affinity between SAMD6 and ALK2. In fact, the regions of SMAD6 C-terminal and UBE2O N-terminal are indispensable for their interaction. UBE2O contains a UBC domain, which has the E2 activity that can induce the monoubiquitination of SMAD6. RNAi or mutation of cysteine 885, which is in UBC domain of UBE2O, attenuates the level of monubiquitinated SMAD6. To be noted, mutated lysine 174 in the region of SMAD6 N-terminal significantly reduced the monoubiquitination levels caused by UBE2O, which implies that UBE2O is mainly responsible for the monoubiquitination of SMAD6 through lysine 174. Moreover, mutation of SMAD6 lysine 174 more obviously inhibited BMP7-induced SMAD1 phosphorylation (Zhang et al., 2013). On the other hand, the expressional level of UBE2O appears to be a positive correlation with BMP7 to promote adipocyte differentiation. HepG2 cells treated with UBE2O-specific shRNAs significantly impaired BMP7-induced signal activation and the phosphorylation level of SMAD (Figure 2). Further studies showed that the nonmodified SMAD6 protein displayed in all subcellular fractions, whereas monoubiquitinated SMAD6 was mainly located in the cytoplasm. These results show that the monoubiquitination of SMAD6 via UBE2O predominantly occurs in the cytoplasm and influences the upstream of SMAD1 phosphorylation. Besides, the catalytically inactive mutant (C885S) of UBE2O is disable to facilitate BMP7-induced SMAD transcriptional responses (Zhang et al., 2013), suggesting that the monoubiquitination of SMAD6 by UBE2O enhances BMP7/SMAD signaling to induce adipocyte differentiation.

## 3. UBE2O inhibits TRAF6-mediated NF-κB activation

Tumor necrosis factor (TNF) receptor-associated factor 6 (TRAF6) has been proved to play a pivotal role in the interleukin-1 receptor (IL-1R) or Toll-like receptor (TLR)-mediated innate immunity (Barton and Medzhitov, 2003; Xing et al., 2017). IL-1R/TLR recognizes ligands such as cytokines IL-1β or pathogen-associated molecular pattern lipopolysaccharides (LPS) to recruit the adaptor proteins IL-1R-associated kinase 1/4 (IRAK1/4) and myeloid differentiation primary response gene 88 (MyD88). Then IRAK1/4 and MyD88 form a complex with TRAF6 and trigger TRAF6 lysine 63 auto-ployubiquitination *via* ubiquitin-conjugating enzyme 13 (UBC13)/Uev1a (Deng et al., 2000). The auto-polyubiquitination of TRAF6 activated TGF-β activated kinase 1 (TAK1) to trigger the degradation of the downstream κ-light-chain enhancer of activated IκBα, which will lead to the phosphorylation of NF-κB, be located to the nucleus and initiate target gene transcriptional responses (Wang et al., 2001). Thus, the polyubiquitination of TRAF6 plays a critical role in NF-κB signal transduction.

**Figure 3:**
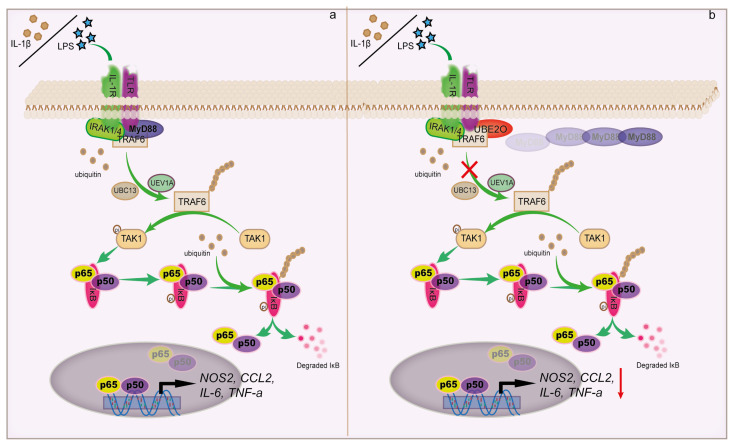
UBE2O inhibits TRAF6-mediated NF-κB activation. a. IL-1β/LPS activates NF-κB signal by inducing TRAF6 auto-ployubiquitination. b. UBE2O replaces the place of MyD88 to prevent the activation of NF-κB that is induced by the polyubiquitinated TRAF-6.

Yeast two-hybrid and coimmunoprecipitation assays demonstrated that the putative E2 enzyme UBE2O interacted with several TRAF family members including TRAF1, TRAF3, TRAF4, TRAF5 and TRAF6. However, UBE2O has the most powerful effects on NF-κB activation that is mediated by TRAF6 (Markson et al., 2009; Zhang et al., 2013). Importantly, UBE2O particularly prevents IL-1β or TLR4-triggered NF-κB activation rather than poly (I: C) or TRIF in a dose-dependent manner. Besides, UBE2O also inhibits the activation of NF-κB reporter induced by MyD88, rather than IKKs or p65. Hence, these results imply that UBE2O functions in the upstream in the TRAF6-mediated NF-κB signaling pathway (Zhang et al., 2013). Overexpression of UBE2O suppresses the IL-1β-induced phosphorylation and degradation of Iκ-Bα, and the expression of NF-κB-targeted genes including IL-6, TNF-α, CCL2 and NOS2 (Figure 3). Consistent with the above, knockdown of UBE2O augments the IL-1β or LPS-induced NF-κB activity and the expression of NF-κB target genes. It should be noted that UBE2O also reduces the phosphorylation of JNK or p38 induced by IL-1β (Zhou et al., 2012). Further investigations demonstrated that UBE2O strongly reduced the polyubiquitination level of TRAF6 whether IL-1β stimulation exists or not. Besides mainly inhibiting the polyubiquitination of K63, UBE2O also blocked the polyubiquitination of TRAF6 K48. Strikingly, the N-terminal UBC domain of UBE2O is dispensable for inhibition of the TRAF6-induced NF-κB activity. The C-terminal deletion mutant (D1) can efficiently interact with TRAF6, which contains a coiled-coil interaction domain but lacks the UBC domain. On the contrary, the N-terminal deletion construct (D2), which contains the UBC domain but lacks the coiled-coil domain, cannot combine with TRAF6. Coimmunoprecipitation analysis by three TRAF6 deletion constructs showed that the C-terminal TRAF domain is indispensable for interaction with UBE2O. What is noteworthy is that the domain is also necessary for the TRAF6 interaction with upstream protein MyD88. Importantly, forced expression of UBE2O impaired the interaction between TRAF6 and MyD88 rather than other upstream proteins, such as the combination between TRAF6 and IRAK1 or MyD88 and IRAK4 (Zhang et al., 2013). These results suggest that UBE2O perhaps plays a role to disturb the interaction between TRAF6 and its regulator MyD88, attenuating TRAF6-polyubiquitination and subsequent NF-κB activity.

## 4. UBE2O mediates BAP1 subcellular location

BRCA1 associated protein-1 (BAP1) is a deubiquitination enzyme that implicates in cell proliferation and tumorigenesis via interaction with chromatin associated proteins (CAPs) (Misaghi et al., 2009; Dey et al., 2012). Recently, it was revealed that UBE2O could be copurified with BAP1, implying that it is likely to be implicated in the regulation of BAP1 function (Sowa et al., 2009; Mashtalir et al., 2014). Mashtalir et al. found that UBE2O not only interacts with wild-type, but also catalytic dead BAP1 (C91S) (Yu et al., 2010). In fact, BAP1 is functionally regulated by several ubiquitination events in the both deubiquitinase (DUB)-activated or nonactivated ways. Investigated several E2s and DUBs demonstrated that UBE2O is specifically directed against ubiquitination of BAP1. The conserved region 1(CR1), conserved region 2 (CR2), and UBC domains of UBE2O are necessary for the ubiquitination of BAP1 because deletion of anyone of them abolishes the ability of UBE2O to bring ubiquitination of BAP1. In vitro and vivo experiments using methylated ubiquitin or K0 ubiquitin mutant, which is unable to form polyubiquitin chains, showed a similar ubiquitination pattern of unmodified ubiquitin. Besides, the proteasome inhibitor MG132 cannot influence the UBE2O-mediated multimonoubiquitination pattern of BAP1 in 293T cells co-transfected with UBE2O and BAP1 or C91S mutant. These results implied that UBE2O catalyzes multimonoubiquitination of BAP1, but is incapable of polyubiquitin chain elongation and subsequent degradation of BAP1 (Mashtalir et al., 2014).

Further studies indicate that the NLS regions of BAP1 including the RRR site on aa 699–701, the hydrophobic GVSIGRL patch on aa 703–709 and the basic amino acid stretch RRKRSRPYKAKRQ on aa 717–729 are indispensable for combination and ubiquitination by UBE2O. MS peptide analysis showed that BAP1 possesses 14 unique ubiquitination sites. Of note, 50% ubiquitination sites are located in the NLS area that occupies only 4% of the whole proteins. Mutation of lysine sites (K/R) in NLS significantly attenuated C91S ubiquitination. Under natural physiological conditions, BAP1 is mainly located in nuclear and UBE2O is predominantly in cytoplasm.

**Figure 4:**
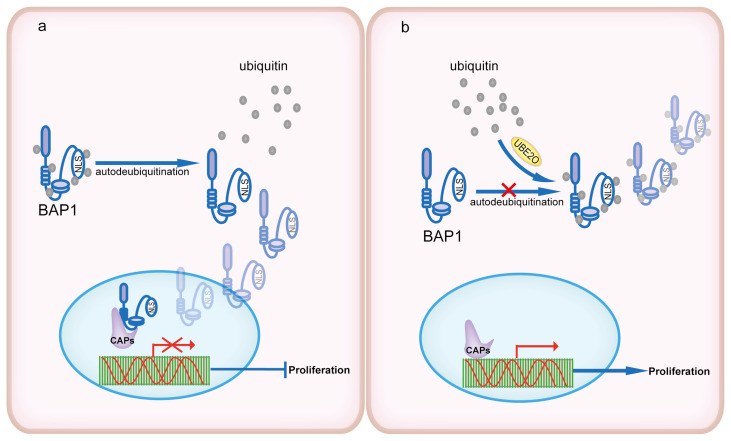
UBE2O mediates BAP1 subcellular location. a. BAP1 maintains the ability of nucleocytoplasmic transport by autodequitination. b. UBE2O regulates BAP1 nucleocytoplasmic transport and subcellular localization and mediates the occurrence of diseases by ubiquitination of the NLS of BAP1.

When coexpressed with UBE2O, BAP1 showed the strong ubiquitin-mediated nuclear export (Figure 4). These results suggest that UBE2O regulates BAP1 nucleocytoplasmic transport and subcellular localization, and mediates the occurrence of diseases by ubiquitination of the NLS of BAP1 (Mashtalir et al., 2014). As a matter of fact, BAP1 contributes to NLS autodeubiquitination against UBE2O ubiquitination by its intramolecular interaction. Mutation of BAP1 disrupts its autodequitination, lending to inappropriate BAP1 localization, which may be another important causes in the occurrence of diseases.

## 5. UBE2O enhances interleukin-1 pathway-induced MLL degradation

Mixed-lineage leukemia (MLL) has been reported to translocate with over 70 partner genes to form diverse oncogenic MLL chimeras, which result in deregulation of target genes and aggressive leukemia (Meyer et al., 2013; Pigneux et al., 2015). These MLL rearrangements mainly occur in pediatric patients and are associated with the poorest prognosis of acute leukemia (Mohan et al., 2010). The MLL chimeras consist of a MLL N-terminal fragment and a C-terminal portion of its partner. Liang et al. found that although wild-type MLL and chimeric MLL have similar mRNA expression, wild-type MLL is much less than the MLL chimeras in the protein level in MLL leukemia cell lines. Therefore, they hypothesized that stability of the wild type MLL protein may replace the MLL chimeras from chromatin and relieve leukemia. Due to that MLL chimeras possess the same N terminal with wild-type MLL, but absent the internal regions in the C-terminal that are responsible for protein breakpoint. These missing regions may be tightly related to the stabilization of MLL. Multidimensional protein identification technology (MudPIT) assay showed UBE2O to be the most abundant protein specifically interacting with MLL internal region (MLL-Inter), which was also confirmed by coimmunoprecipitation. The UBE2O could not bind the most common MLL chimeras including MLL-ENL, MLL-AF9, MLL-AFF1 and MLL-ELL (Liang et al., 2017). In fact, the first PHD finger domain and the region spanning the MLL breakpoint region are necessary and adequate for the interaction between MLL and UBE2O.

Overexpression of UBE2O significantly induced proteasome-mediated MLL degradation in HEK293 cells. In agreement, knockdown of UBE2O by two independent shRNAs increased wild-type MLL protein levels. Further studies demonstrated that IL-1β markedly induces the MLL degradation and promotes the interaction of UBE2O and MLL-Inter. Ectopic expression of UBE2O increases the ubiquitination of MLL-Inter, which could be further enhanced by the stimulation of IL-1β. Moreover, IRAK4 could directly phosphorylate UBE2O and deletion of IRAK4 or treatment with IRAK4 inhibitor represses the MLL-UBE2O interaction, indicating that IL-1β/IRAK4/UBE2O signaling mediates the degradation of MLL. Deletion of UBE2O or cells treated with IRAK4 inhibitor result in a greater inhibition in MLL-dependent (SEM) cell proliferation, compared to non-MLL (REH) cells. In fact, overexpression of the MLL N-terminus of MLL, which cannot bind UBE2O but contains chromatin-binding domains, reduced the proliferation of SEM cells, indicating that destabilizing wild-type MLL is prerequisite for the proliferation of MLL leukemia cells (Liang et al., 2017). Inhibition of IRAK4 to regulate UBE2O markedly increases the chromatin occupation of wild-type and inhibits the proliferation of MLL leukemia, which provides us a paradigm to develop a novel therapy method for aggressive MLL leukemia and other cancers caused by improper translocation.

## 6. UBE2O promotes activation of the mTOR-HIF1a pathway by the ubiquitination degradation of AMPKa2

Recent studies showed that UBE2O is amplified in several human cancers including liver, beast, bladder and lung carcinoma, indicating UBE2O may exert a positive effect on cancer cell survival (Briffa et al., 2015; Vila et al., 2017). Moreover, classical focus formation assays by adenovirus H-RasV12 and E1A revealed Ube2o^−/−^ mouse embryonic fibroblasts (MEFs) have lesser number of foci of morphologically transformed cells than wild-type cells, indicating that UBE2O promotes cellular oncogenic transformation. To examine the function of UBE2O in vivo, Vila and his colleagues crossbred the Ube2o knockout mouse with two transgenic mouse models of spontaneous cancer (MMTV-PyVT mice for breast cancer and TRAMP mice for prostate cancer). UBE2O ablation in MMTV-PyVT mice markedly inhibits breast cancer initiation, progression, angiogenesis and lung metastasis. Similarly, knockout of UBE2O in TRAMP mice improves prostate cancer survival, invasion and metastasis. These results suggest that UBE2O might be a new therapeutic target for cancer.

Mechanistic researches showed that UBE2O has the ability to bind AMPKa2 protein leading to ubiquitination on its lysine-470 (K470) residue, which is a conserved residue in AMPKa2 of vertebrate species but substituted by an arginine (R475) in AMPKa1. UBE2O promoted K48-linked ubiquitination degradation of AMPKa2 in vitro and in vivo, but not AMPKa1 (Vila et al., 2017). Furthermore, knockdown of UBE2O increased AMPKa2 protein levels, leading to a strong activation of its downstream target genes. Ube2o ablation in two mouse models of lymphoma (an Em-Myc mouse model of B cell lymphoma and a Pten-deficient mouse developing T cell lymphoma) in which AMPKa2 is undetectable, did not produce a markedly antitumor effect, suggesting that AMPKa1 might not play a role in UBE2O-dependent tumorigenesis (Vila et al., 2017). Further studies showed that UBE2O promoted the activation of the rapamycin complex 1 (mTORC1) signaling and downstream hypoxia-inducible factor 1-a (HIF1a) through S6 phosphorylation in an AMPKa2-dependent manner. The test of UBE2O inhibitor ATO in mouse models of breast and prostate cancers demonstrated that it can reduce tumor incidence and progression, and extend mouse survival (Vila et al., 2017). Hence, these results indicate that UBE2O is a new cancer therapeutic target, which modulates the AMPKα2-mTOR-HIF1α axis.

## 7. UBE2O induces myeloma cell apoptosis *via* modulating c-Maf stability

Transcription factor c-Maf is a member of Maf family, which highly expresses in over 50% of primary multiple myeloma (MM) patient samples and MM cell lines. c-Maf significantly enhances myeloma cell proliferation in vitro and tumor formation in vivo. c-Maf transgenic mice display the MM-like symptoms. From these results c-Maf has been considered as an important regulator to the MM, which could be a target for MM therapy (Hurt et al., 2004).

Zhang et al. discovered that c-Maf goes through degradation by the ubiquitin proteasome pathways and destabilizing c-Maf inhibits the growth of MM xenografts in mice, but its ubiquitin conjugating enzyme is not clear (Zhang et al., 2016). LC/MS/MS assay to analyze the c-Maf specific antibody affinity products demonstrated that UBE2O is a unique ubiquitin conjugating enzyme, which is also proved by immunoprecipitation. The ubiquitination assay in tube revealed that UBE2O markedly increases the level of c-Maf ubiquitination without an extra E3 ligase. Moreover, cotransfected c-Maf and UBE2O with the mutated ubiquitin plasmid K48R or K63R in HEK293T cells revealed that the K63R-Ub mediated the ubiquitination of c-Maf, but not K48R, indicating that UBE2O mediates the polyubiquitination c-Maf at K48. On the other hand, MG132 treatment abolishes the degradation of c-Maf protein that is mediated by UBE2O. These results suggested that UBE2O mediates the polyubiquitination of c-Maf at K48 and degradation in the proteasome pathway. It was found by a series of mutants of lysine (K) to arginine (R) that the K331 and K345 residues of c-Maf were necessary for interacting with UBE2O. Further researches illustrated that UBE2O induced MM cell apoptosis and suppressed cell proliferation both in vitro and in vivo, which is associated with the c-Maf expression protein level. Based on the above results, UBE2O mediating c-Maf stability may be a novel insight to myeloma therapy.

## 8. Conclusion

UBE2O participates in regulating the degradation of orphans of multiprotein complexes, hereby influencing occurrence of diseases. It binds SMAD6/SMAD7 to partially contribute to BMP/SMAD signal regulation to induce adipocyte differentiation. UBE2O can disturb the interaction between TRAF6 and its regulator MyD88, attenuating TRAF6-polyubiquitination and subsequent NF-κB activity. UBE2O also regulates BAP1 nucleocytoplasmic transport and subcellular localization to mediate the occurrence of diseases by ubiquitination of the NLS of BAP1. It induces myeloma cell apoptosis *via* modulating c-Maf stability and is intimately associated with occurrence of esophageal and breast cancer due to the deficiency of 17q25.1, possibly becoming a new cancer therapeutic target through modulating the AMPKa2-mTOR-HIF1a axis. UBE2O may affect endosomal protein trafficking and cell proteome through retromer-mediated transportation and endosomal F-actin (Hao et al., 2013). It also recognizes ribosomal proteins and other substrates to induce erythroid proteome to undergo a rapid and drastic change at the reticulocyte stage (Nguyen et al., 2017). Importantly, UBE2O has been suggested as a quality control supervisor of multiprotein complexes for degradation (Yanagitani et all., 2017). Hence, the progression on UBE2O provides a new perspective to develop drugs by regulating UBE2O activity. However, the current understanding of UBE2O is quite limited and the detailed regulatory mechanisms remain to be unclear. Although it is revealed that UBE2O exerts the E3 activity in N-terminal portion, further researches need to identify the cysteine residue which functions as an E3 active site. Besides its E2–E3 activity, other actions of UBE2O merit to be explored as well. Together, UBE2O plays a vital role in intracellular protein ubiquitination regulation. Elucidating the functions of UBE2O may lead to identification of neoteric crucial molecular targets so as to pave therapeutic approaches for ubiquitination-associated metabolic disorders and diseases.

## Figures and Tables

**Table:** Signal pathways and functions of UBE2O.

Substrate	Pathway	Function	Reference
WASH	WASH/F-actin	Promoting retrograde transport	[33]
SMAD6	BMP7/SMAD6	Inducing adipocyte differentiation	[7]
TRAF6	TRAF6/NF-κB	Preventing the activation of NF-κB by IL-1β and LPS	[11]
BAP1	Indetermination	Regulating BAP1 subcellular localization and disturbing cell proliferation	[9]
MLL	Indetermination	Disturbing stability of MLL and augmenting MLL leukemia	[6]
AMPKa2	AMPKa2/mTOR/HIF1a	Facilitating breast and prostate cancers	[10]
c-Maf	Indetermination	Promoting c-Maf degradation and depressing multiple myeloma	[8]
BMAL1	Indetermination	Modulating circadian rhythm	[35]
RPs	Indetermination	Remodeling erythroid proteome	[37]
Non-RPs	Indetermination	Indetermination	[37]

## References

[b1-turkjbiol-46-2-186] Barton GM Medzhitov R 2003 Toll-like receptor signaling pathways Science 300 5625 1524 1525 12791976 10.1126/science.1085536

[b2-turkjbiol-46-2-186] Briffa R Um I Faratian D Zhou Y Turnbull AK 2015 Multi-Scale Genomic, Transcriptomic and Proteomic Analysis of Colorectal Cancer Cell Lines to Identify Novel Biomarkers PLoS One 10 12 e0144708 26678268 10.1371/journal.pone.0144708 PMC4692059

[b3-turkjbiol-46-2-186] Cai J Culley MK Zhao Y Zhao J 2018 The role of ubiquitination and deubiquitination in the regulation of cell junctions Protein Cell 9 9 754 769 29080116 10.1007/s13238-017-0486-3 PMC6107491

[b4-turkjbiol-46-2-186] Deng L Wang C Spencer E Yang L Braun A 2000 Activation of the IkappaB kinase complex by TRAF6 requires a dimeric ubiquitin-conjugating enzyme complex and a unique polyubiquitin chain Cell 103 2 351 361 11057907 10.1016/s0092-8674(00)00126-4

[b5-turkjbiol-46-2-186] Dey A Seshasayee D Noubade R French DM Liu J 2012 Loss of the tumor suppressor BAP1 causes myeloid transformation Science 337 6101 1541 1546 22878500 10.1126/science.1221711 PMC5201002

[b6-turkjbiol-46-2-186] Hao YH Doyle JM Ramanathan S Gomez TS Jia D 2013 Regulation of WASH-dependent actin polymerization and protein trafficking by ubiquitination Cell 152 5 1051 1064 23452853 10.1016/j.cell.2013.01.051 PMC3640276

[b7-turkjbiol-46-2-186] Hormaechea-Agulla D Kim Y Song M Song S 2018 New Insights into the Role of E2s in the Pathogenesis of Diseases: Lessons Learned from UBE2O Molecules and Cells 41 3 168 178 29562734 10.14348/molcells.2018.0008 PMC5881090

[b8-turkjbiol-46-2-186] Hurt EM Wiestner A Rosenwald A Shaffer AL Campo E 2004 Overexpression of c-maf is a frequent oncogenic event in multiple myeloma that promotes proliferation and pathological interactions with bone marrow stroma Cancer Cell 5 2 191 199 14998494 10.1016/s1535-6108(04)00019-4

[b9-turkjbiol-46-2-186] Itoh S ten Dijke P 2007 Negative regulation of TGF-beta receptor/Smad signal transduction Current Opinion in Cell Biology 19 2 176 184 17317136 10.1016/j.ceb.2007.02.015

[b10-turkjbiol-46-2-186] Klemperer NS Berleth ES Pickart CM 1989 A novel, arsenite-sensitive E2 of the ubiquitin pathway: purification and properties Biochemistry 28 14 6035 6041 2550069 10.1021/bi00440a047

[b11-turkjbiol-46-2-186] Liang K Volk AG Haug JS Marshall SA Woodfin AR 2017 Therapeutic Targeting of MLL Degradation Pathways in MLL-Rearranged Leukemia Cell 168 1–2 59 72e13 28065413 10.1016/j.cell.2016.12.011 PMC5351781

[b12-turkjbiol-46-2-186] Markson G Kiel C Hyde R Brown S Charalabous P 2009 Analysis of the human E2 ubiquitin conjugating enzyme protein interaction network Genome Research 19 10 1905 1911 19549727 10.1101/gr.093963.109 PMC2765280

[b13-turkjbiol-46-2-186] Mashtalir N Daou S Barbour H Sen NN Gagnon J 2014 Autodeubiquitination protects the tumor suppressor BAP1 from cytoplasmic sequestration mediated by the atypical ubiquitin ligase UBE2O Molecules and Cells 54 3 392 406 10.1016/j.molcel.2014.03.002 24703950

[b14-turkjbiol-46-2-186] Meyer C Hofmann J Burmeister T Groger D Park TS 2013 The MLL recombinome of acute leukemias in 2013 Leukemia 27 11 2165 2176 23628958 10.1038/leu.2013.135 PMC3826032

[b15-turkjbiol-46-2-186] Misaghi S Ottosen S Izrael-Tomasevic A Arnott D Lamkanfi M 2009 Association of C-terminal ubiquitin hydrolase BRCA1-associated protein 1 with cell cycle regulator host cell factor 1 Molular and Cellular Biology 29 8 2181 2192 10.1128/MCB.01517-08 PMC2663315 19188440

[b16-turkjbiol-46-2-186] Miyazono K Kamiya Y Morikawa M 2010 Bone morphogenetic protein receptors and signal transduction Journal of Biochemistry 147 1 35 51 19762341 10.1093/jb/mvp148

[b17-turkjbiol-46-2-186] Mohan M Lin C Guest E Shilatifard A 2010 Licensed to elongate: a molecular mechanism for MLL-based leukaemogenesis Nature Reviews Cancer 10 10 721 728 10.1038/nrc2915 20844554

[b18-turkjbiol-46-2-186] Nguyen AT Prado MA Schmidt PJ Sendamarai AK Wilson-Grady JT 2017 UBE2O remodels the proteome during terminal erythroid differentiation Science 357 eaan0218 28774900 10.1126/science.aan0218 PMC5812729

[b19-turkjbiol-46-2-186] Pigneux A Labopin M Maertens J Cordonnier C Volin L 2015 Outcome of allogeneic hematopoietic stem-cell transplantation for adult patients with AML and 11q23/MLL rearrangement (MLL-r AML) Leukemia 29 12 2375 2381 26082270 10.1038/leu.2015.143

[b20-turkjbiol-46-2-186] Plouhinec JL Zakin L De Robertis EM 2011 Systems control of BMP morphogen flow in vertebrate embryos Current opinion in genetics and development 21 6 696 703 21937218 10.1016/j.gde.2011.09.001 PMC3224208

[b21-turkjbiol-46-2-186] Sieber C Kopf J Hiepen C Knaus P 2009 Recent advances in BMP receptor signaling Cytokine Growth Factor Review 20 5–6 343 355 10.1016/j.cytogfr.2009.10.007 19897402

[b22-turkjbiol-46-2-186] Sowa ME Bennett EJ Gygi SP Harper JW 2009 Defining the human deubiquitinating enzyme interaction landscape Cell 138 2 389 403 19615732 10.1016/j.cell.2009.04.042 PMC2716422

[b23-turkjbiol-46-2-186] Tsuchida S Satoh M Takiwaki M Nomura F 2017 Ubiquitination in Periodontal Disease: A Review International Journal of Molecular Science 18 7 1476 10.3390/ijms18071476 PMC5535967 28698506

[b24-turkjbiol-46-2-186] Ullah K Zubia E Narayan M Yang J Xu G 2019 Diverse roles of the E2/E3 hybrid enzyme UBE2O in the regulation of protein ubiquitination, cellular functions, and disease onset The FEBS Journal 286 11 2018 2034 30468556 10.1111/febs.14708

[b25-turkjbiol-46-2-186] van Wijk SJ Timmers HT 2010 The family of ubiquitin-conjugating enzymes (E2s): deciding between life and death of proteins Federation of American Societies for Experimental Biology 24 4 981 993 10.1096/fj.09-136259 19940261

[b26-turkjbiol-46-2-186] Vila IK Song SJ Song MS 2017 A new duet in cancer biology: AMPK the typical and UBE2O the atypical Molecular & Cellular Oncology 4 3 e1304846 28616582 10.1080/23723556.2017.1304846 PMC5462509

[b27-turkjbiol-46-2-186] Vila IK Yao Y Kim G Xia W Kim H 2017 A UBE2O-AMPKalpha2 Axis that Promotes Tumor Initiation and Progression Offers Opportunities for Therapy Cancer Cell 31 2 208 224 28162974 10.1016/j.ccell.2017.01.003 PMC5463996

[b28-turkjbiol-46-2-186] Wang C Deng L Hong M Akkaraju GR Inoue J 2001 TAK1 is a ubiquitin-dependent kinase of MKK and IKK Nature 412 6844 346 351 11460167 10.1038/35085597

[b29-turkjbiol-46-2-186] Xing Y Yao X Li H Xue G Guo Q 2017 Cutting Edge: TRAF6 Mediates TLR/IL-1R Signaling-Induced Nontranscriptional Priming of the NLRP3 Inflammasome Journal of Immunology 199 5 1561 1566 10.4049/jimmunol.1700175 28739881

[b30-turkjbiol-46-2-186] Xu Y Zhang Z Li J Tong J Cao B 2017 The ubiquitin-conjugating enzyme UBE2O modulates c-Maf stability and induces myeloma cell apoptosis Journal of Hematologyand Oncology 10 1 132 10.1186/s13045-017-0499-7 PMC5496436 28673317

[b31-turkjbiol-46-2-186] Yanagitani K Juszkiewicz S Hegde RS 2017 UBE2O is a quality control factor for orphans of multiprotein complexes Science 357 6350 472 475 28774922 10.1126/science.aan0178 PMC5549844

[b32-turkjbiol-46-2-186] Yokota T Nagai H Harada H Mine N Terada Y 2001 Identification, tissue expression, and chromosomal position of a novel gene encoding human ubiquitin-conjugating enzyme E2-230k Genetics 267 1 95 100 10.1016/s0378-1119(01)00407-3 11311559

[b33-turkjbiol-46-2-186] Yu H Mashtalir N Daou S Hammond-Martel I Ross J 2010 The ubiquitin carboxyl hydrolase BAP1 forms a ternary complex with YY1 and HCF-1 and is a critical regulator of gene expression Molecular and Cellular Biology 30 21 5071 5085 20805357 10.1128/MCB.00396-10 PMC2953049

[b34-turkjbiol-46-2-186] Zhang X Zhang J Bauer A Zhang L Selinger DW 2013 Fine-tuning BMP7 signalling in adipogenesis by UBE2O/E2-230K-mediated monoubiquitination of SMAD6 EMBO Journal 32 7 996 1007 23455153 10.1038/emboj.2013.38 PMC3616286

[b35-turkjbiol-46-2-186] Zhang X Zhang J Zhang L van Dam H ten Dijke P 2013 UBE2O negatively regulates TRAF6-mediated NF-kappaB activation by inhibiting TRAF6 polyubiquitination Cell Research 23 3 366 377 23381138 10.1038/cr.2013.21 PMC3587711

[b36-turkjbiol-46-2-186] Zhang Z Tong J Tang X Juan J Cao B 2016 The ubiquitin ligase HERC4 mediates c-Maf ubiquitination and delays the growth of multiple myeloma xenografts in nude mice Blood 127 13 1676 1686 26825710 10.1182/blood-2015-07-658203

[b37-turkjbiol-46-2-186] Zhou F Zhang X van Dam H Ten Dijke P Huang H Zhang L 2012 Ubiquitin-specific protease 4 mitigates Toll-like/interleukin-1 receptor signaling and regulates innate immune activation Journal of Biological Chemistry 287 14 11002 11010 22262844 10.1074/jbc.M111.328187 PMC3322833

